# Extraction of Oleic Acid from Moroccan Olive Mill Wastewater

**DOI:** 10.1155/2016/1397852

**Published:** 2016-01-10

**Authors:** Reda Elkacmi, Noureddine Kamil, Mounir Bennajah, Said Kitane

**Affiliations:** ^1^Department of Chemistry and Valorisation, Faculty of Sciences Ain-Chock, HASSAN II University of Casablanca, BP 5366 Maarif, Casablanca, Morocco; ^2^Process Engineering and Environment Laboratory (PEEL), High School of Technology of Casablanca, HASSAN II University of Casablanca, BP 8012 Oasis, Casablanca, Morocco; ^3^Department of Process Engineering, National School of Mineral Industries of Rabat, BP 753 Agdal, Rabat, Morocco

## Abstract

The production of olive oil in Morocco has recently grown considerably for its economic and nutritional importance favored by the country's climate. After the extraction of olive oil by pressing or centrifuging, the obtained liquid contains oil and vegetation water which is subsequently separated by decanting or centrifugation. Despite its treatment throughout the extraction process, this olive mill wastewater, OMW, still contains a very important oily residue, always regarded as a rejection. The separated oil from OMW can not be intended for food because of its high acidity of 3.397% which exceeds the international standard for human consumption defined by the standard of the Codex Alimentarius, proving its poor quality. This work gives value addition to what would normally be regarded as waste by the extraction of oleic acid as a high value product, using the technique of inclusion with urea for the elimination of saturated and unsaturated fatty acids through four successive crystallizations at 4°C and 20°C to have a final phase with oleic acid purity of 95.49%, as a biodegradable soap and a high quality glycerin will be produced by the reaction of saponification and transesterification.

## 1. Introduction

The production of Moroccan olive oil has been growing and its consumption has also increased (3.9 kg/inhabitant) in 2013 [1], thanks to its nutritional, medical, and economic importance; it participates with 5% in the Moroccan agricultural GDP and 15% in agrifood exports [2]. Morocco is one of the Mediterranean countries concerned with the attractive developing production of olive oil, with an annual production capacity of 1.5 million tons of olives (amount of 0.6 million tons is triturated by about 565 modern units and semimodern ones and amount of 0.16 million tons of olives per year is triturated by 15,000 traditional units called maâsra) [3]. Alongside their activities, these traditional factories produce solid waste called pomace, mainly used in composting [4], combustible [5], biogas [6], tanning [7], or animal feed [8], and also liquid waste called “Olive Oil Mill Wastewater (OMW),” a variation amount between 0.5 and 1.5 m^3^ per 1 ton of olives according to the production method [9]. The “vegetation water” is sent directly to the environmental medium which poses a serious environmental problem because it contains in addition to the acidic pH significant quantities of organic matter and poorly biodegradable polyphenols.

The composition of this olive mill wastewater varies depending on several factors such as the variety and maturity of the olives, the period of production, the climatic conditions, farming methods, and the oil extraction mode [10]. The main physicochemical characteristics of the olive mill wastewater of the region of Fes Boulemane [11] are given in Table 1.

The physicochemical characterization of OMW of the region of Fes Boulemane shows that this effluent has an acid pH value, with a very high chemical oxygen demand (COD) which proves that the OMW constitutes an important environmental problem.

Many biotechnological applications have been made to utilize these liquids that we mentioned; the most commonly used application shown is as follows.

*(1) The Lagooning*. This natural purification process reduces the load rejection in organic matter and polyphenols existing in the olive mill wastewater, to obtain treated water that meets the physicochemical quality standard; it is based on the collection of OMW in ponds outdoors. The organic matter is degraded under the effect of the biological activity of microorganisms, leading to water denitrification [12–15].

This method has the disadvantages of excessive area requirement, the release of bad odors, and the infiltration of pollutants in the basement to land groundwater.

*(2) The Composting*. Composting of OMW is a technique used to improve the physical, chemical, and biological properties of soil. It is based on the decomposition of organic matter into stable products rich in humic compounds.

Several studies on composting of OMW were conducted [16–18]. This technique improves the water retention in sandy soils and aggregate stability and the cation exchange capacity, which increases microbial activity, and promotes the degradation of pesticides and other organic compounds [19]. 

*(3) Use as Fertilizer*. Due to environmental restrictions of the lagooning process of OMW, it can be used as a fertilizer. The high organic load and the concentration of soluble nutrients gives it wide use in agriculture [20, 21].

But its high pollution load, toxicity, and transport costs may limit the use of OMW as fertilizers. 

*(4) Use as Animal Feed*. The high content of sodium and phenolic compounds in the OMW causes digestive disorders in ruminants [22]. Concerning this problem, research has therefore focused on reducing phenols by specific processes. Dalmolive process [23, 24] reduces phenol to an acceptable tenor. 29 kg of food can be produced by combining 50 kg of OMW with 20 kg of exhausted pomace and 12.6 kg of agricultural derivatives. 

*(5) Biogas Production*. The anaerobic digestion process is based on the biochemical conversion of organic matter to produce carbon dioxide and methane [25–27].

A volume of 1 m^3^ of OMW contains a concentration of 70 kg of chemical oxygen demand (COD) producing about 24.5 m^3^ of methane. The energy of the biogas is used in thermal form and can be converted into electrical energy [28].

Physicochemical and electrochemical processes were used to treat this effluent in order to reduce the organic matter and toxicity to acceptable limits such as lime treatment [29, 30], coagulation-flocculation-hydrogen peroxide oxidation [31], and phenolic treatment [32–34].

The principal aim of the present work was to develop a simple and easy method to recover valuable products from these effluents, discharged directly into the environment with huge amount without effective treatment.

The originality of this work lies in the separation by a natural setting of oil from olive mill wastewater, and after several analyses of this separated oil, we found its acidity higher than human consumption standard, which requires searching recovery solutions by adaptation and application of a fractional extraction technique of fatty acid from these effluents for their valuation coupled on one hand to produce soap and glycerin and on the other hand to extract pure oleic acid contained in this extracted oil.

## 2. Materials and Methods

After the extraction of olive oil by pressing or centrifuging, the resulting liquid contains oil and vegetation water; the latter is separated by decanting or centrifugation.

Despite their treatment, the rejects from decantation and centrifuging still contain very important oily residues, usually discarded in the environmental media.

The residual oil after separation of OMW may not be used for consumption for its high acidity (it reaches about 3.397%), value exceeding the Codex Alimentarius standard [35].

In our work, we collected 90 L of OMW in the region of Fes Boulemane and after storage in cans of 5 liters, they are preserved in the laboratory for six months at room temperature for decantation.

### 2.1. Olive Mill Wastewater Characterization

Olive mill wastewater used in this work was collected from diverse traditional crushing units of Fes Boulemane region, during the olive oil year 2012/2013.

The source and mass fraction purity of materials are listed in Table 2.

### 2.2. Analyses

#### 2.2.1. Oil Analysis

The chemical characterization of recovered oil samples and the soap product was performed according to the method of the International Organization for Standardization (ISO) [36–46].

#### 2.2.2. Samples Analysis by GC

The gas chromatography can be applied directly to fatty acids and fatty esters. Regarding triglycerides, they are used for the study of chain length after conversion into methyl esters.

During separation, samples were analyzed using a gas chromatography having the following characteristics:

 Name: VARIANT 304 CX.
 Column length: 50 m.
 Stationary phase: silica.
 Carrier gas: He.
 
*T* column: 210°C.
 Detector: FID (Flame Ionization Detector).

#### 2.2.3. Determining Density

For measuring the density, our sample is weighed with a balance and then placed into a graduated cylinder filled with 100 mL of water.

The elevation value of water volume in the graduated cylinder has allowed us to calculate the value of the density.

#### 2.2.4. Determining Boiling Point

To characterize our resultant products we determined their boiling point, by placing the samples (oleic acid, glycerol) in a test tube and a thermometer in place, after heating with a hotplate until the appearance of the first bubble of vapor, the boiling temperature was determined by the thermometer at atmospheric pressure.

#### 2.2.5. Determining Melting Point

The Thiele tube is used to determine the melting point of our products, the sample is placed in a capillary connected with a thermometer and immersed in the tube, and after heating the product began to melt where we note the melting temperature.

### 2.3. Extraction of Oleic Acid

Oleic acid ((9Z)-octadec-9-enoic acid) is an unsaturated fatty acid. This essential compound of chemistry is also used as a surfactant to modify the surface of magnetite particles [47], in the pharmaceutical field [48], and is considered as a raw material for the production of bioproducts little available in nature [49].

Several techniques have been developed to extract the oleic acid from food waste; the most frequently used one is based on the fractional distillation [50, 51] and the method of inclusion with urea [52, 53]; it remains more advantageous not only because of its lower cost and its better yield, but also because of the quality of the recovered acid, as well as the low temperature adopted for the extraction protecting oleic acid oxidation.

In this work, the method based on the fractional crystallization of urea described by Frémont and Gozzelino [54] was adopted. It thus permits the recovery of a highly purified oleic acid with a very good quality; the difference was that they worked with olive oil; then we can recover this acid by OMW.

#### 2.3.1. Transesterification

The aim of this technique is to transform the triglycerides which constitute the oil, to methyl esters. For this purpose, 200 mL of this oil sample was mixed with 600 mL of methanol. The reaction is catalyzed by 100 mL of sodium methoxide (prepared before by mixing of 1 g of sodium hydroxide with 100 mL of methanol). The mixture is subjected to heating under reflux for 3 hours at 60°C period considered sufficient for a perfect homogenization of the mixture.

The mixture is then separated in a separatory funnel for 4 hours until the appearance of two phases: a top layer rich in methyl ester and a lower layer rich in glycerol. After the recovery of the latter, the separated upper phase is washed properly with hydrochloric acid to neutralize excess sodium hydroxide.

This upper phase of the first separation is rewashed a second time with 100 mL of distilled water, and to form two layers, the upper one rich in pure methyl esters and the lower one rich in water and methanol.

After separating the esters from the upper phase, the lower phase is thus introduced into a separatory funnel in the presence of hexane. A three-stage separation is performed to maximize recovery of methyl esters, successive additions of hexane were made at a rate of 25 mL, and each addition is followed by a separation by decantation. After the adding of the pure methyl esters recovered in the first separation to that extracted by hexane, the mixture was concentrated in the rotatory evaporator, and the esters thus purified were also weighed with an electronic scale.

#### 2.3.2. Crystallization with Urea

The objective of this step is to crystallize methyl oleate by the four successive crystallizations with a purity of about 95. 5% from the methyl esters of fatty acids prepared.

For a first separation, we take 100 g of methyl esters mixed with 100 g of urea and 1 L methanol, using a water bath to achieve solubilization in alcohol.

After cooling overnight at 4°C, our solution is filtered on sintered glass, under vacuum of 1 bar maintained with a vacuum pump (KNF NEUBERGER), trying to keep the same conditions to obtain two phases solid C1 and filtrate F1.

The filtrate F1 is mixed with 200 g of urea. The mixture was maintained at 4°C, to have C2 crystals that will be rinsed and mixed with 1.5 L of methanol and filtered under the vacuum at room temperature.

Filtrate F3 product of the second crystallization is subjected to a temperature of 4°C after adding 120 g of urea. The mixture is allowed to rest until the appearance of crystalline phase C4.

This final phase additionally contains methyl esters urea which must be removed by a hydrochloric acid solution and separated after a natural decantation. These esters are then extracted with hexane, and the excess of acid was removed by pure water and then dried over anhydrous sodium sulfate.

After the removal of the solvent with the rotary evaporator, we weighed resulting esters.

### 2.4. Saponification

For soap from OMW fast saponification is performed, using a strong base in reflux mounting [55].

#### 2.4.1. Implementation of the Saponification Reaction

A volume of 150 mL of oil recovered from OMW is mixed with 150 mL of ethanol in the presence of 30% sodium hydroxide as an alkaline agent without forgetting a few grains of pumice.

The mixture was kept heating under reflux for 4 hours at 50°C, period deemed sufficient enough for the completion of the saponification reaction until the solution becomes limpid, and our mixture is allowed to settle for a few minutes. The reaction mixture is then separated into two phases, an aqueous one rich in glycerin and a second heavier one which is soap.

The reaction mixture was treated with a solution containing 200 g/L of sodium chloride; the neutralization of NaOH significantly improves the separation of the two phases.

#### 2.4.2. Separation by Filtration

The reaction mixture undergoes a night of natural decantation, for 12 hours. Then, it was vacuum filtered by suction on Büchner funnel through a filter cloth (40 *μ*m). The recovered solid phase (soap) was washed two times with 50 mL of distilled water and dried by sunshine.

### 2.5. Extraction of Glycerin

Glycerol (propane-1,2,3-triol) is a coproduct which has three hydroxyl groups, functionalisable and used in food [56, 57] and cosmetics [58, 59]; there are two types of glycerol, a synthetic one obtained by the petrochemical process in which propene is converted to glycerol and natural one formed in the two processes of saponification and transesterification [60].

After separation of the aqueous alkaline phase, we will neutralize it with concentrated hydrochloric acid at 12%. After a simple distillation followed by evaporation we can isolate the glycerin (or glycerol).

## 3. Results and Discussion

The oil of OMW was recovered by natural decantation for six months; the 90 liters gave 20 liters (yield 23%). This value is slightly higher proving that despite these effluents treatment remains rich in oil. The characteristics of this oil are shown in Table 3.

Examination of the recovered oil characteristics has shown that the acidity is greater than that for the Codex Alimentarius standard due to lack of oil stability, bad storage conditions, and low peroxide index explained by resistance to oxidation during storage.

**Figure 1:**
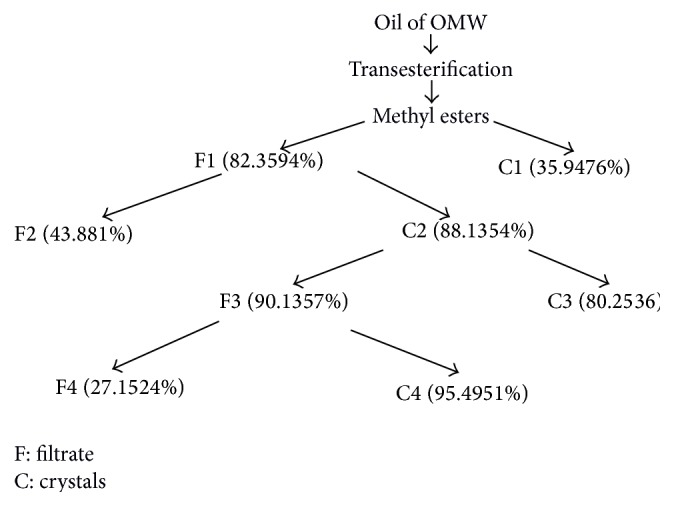
Fractional crystallization steps.

After a series of fractional crystallizations, we could have in the end crystallized phase C4 rich in methyl oleate with a purity of 95.49% (Figure 1).

We find that during these four successive separations most of oleic acid either in the crystal or in the filtrate depends on the temperature conditions and dilution.

The majority of saturated fatty acids are eliminated in the first separation at a temperature of 4°C, in the same condition and by adding the urea to the filtrate F1; the rest will be eliminated in the second crystallization, which gives rise to phase C2 having in addition to the oleic acid an amount of saturated fatty acid.

This phase will be diluted with methanol at 20°C to prevent the formation of inclusions, with the elimination of fatty acids, and recover oleic acid in filtrate F3 with 90.1357% purity.

A final crystallization gave us by the addition of urea at 4°C a final phase rich in oleic acid (95.4951%).

The evolution in the composition of fatty acids in the four crystallizations is mentioned in Table 4.

In the first separation we could eliminate the saturated acids C16:0 (palmitic acid) and C18:0 (stearic acid) with significant fractions 38.8984% and 13.9547%, respectively. After the second crystallization we could recover in filtrate F2 some of these saturated acids and a part of the unsaturated acids including oleic acid.

C2 crystals contain a small amount of saturated acids which will be crystallized in the third separation where we have used a large amount of methanol at room temperature to form inclusions with these acids. We have also seen a significant distribution of our oleic acid in both phases C3 (80.2536%) and F3 (90.1357%).

In the fourth crystallizing filtrate F3 gave birth to crystalline phase C4 with a highly pure oleic acid and (2.5164%) of palmitic acid and traces of other unsaturated acids.

We can separate oleic acid, using saponification followed by addition of hydrochloric acid, until the occurrence of an oleic acid precipitate whose characteristics are presented in Table 5.

The resulting oleic acid has a yellow color; it is insoluble in water and soluble in some solvent such as ethanol, ether, and chloroform; this product is used to prepare esters, alcohols, and organometallic salt.

With the quality and purity of this compound, we can produce biodiesel by esterification with alcohol.

**Figure 2:**
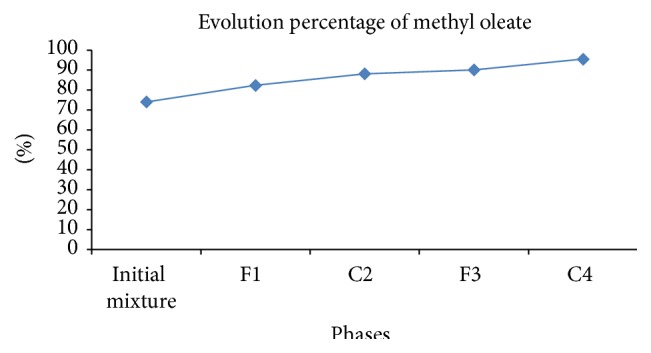
Composition of methyl oleate in phases during crystallization.

The oxidative cleavage of the acid produces two unsaturated carboxylic acids: azelaic acid (or acid nonanedioic) used in the cosmetic and pharmaceutical field (treatment of skin diseases including acne) and pelargonic acid (or nonanoic acid) frequently used as fragrance for perfumes, antibacterial, surfactants, and others [61, 62].  Figure 2 shows the variation of the composition of methyl oleate during the stages of crystallization.

The initial mixture (separated oil) contains 74.018% methyl oleate, and after the first crystallization we are left with a percentage of 82.3594%; C2 crystals resulting from the second separation have a purity of 88.1354%. F3 and C4 contain 90.1357% and 95.495%, respectively. Frémont et al. were able to extract an oleic acid with purity of 99.5% but by starting from an olive oil and cooling to −60°C of final phase C4 mixed with an acetone solution.

The purity of our oleic acid is of the order of 95.4951%, which is acceptable because we are working with olive mill wastewater instead of virgin olive oil.

**Figure 3:**
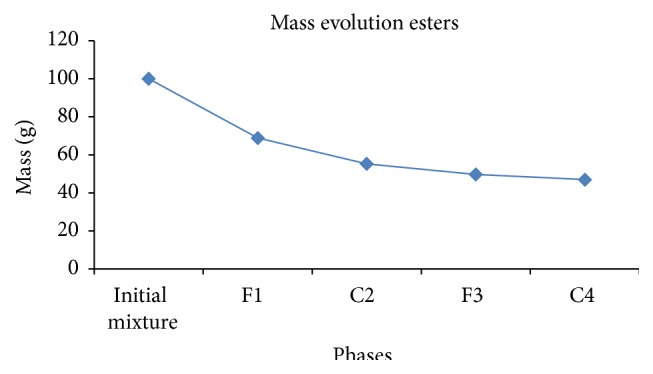
Evolution esters mass during crystallization.

Transesterification of 200 mL oil gave approximately 180.65 g of methyl esters where 100 g is used for crystallization. After separating the four were able to get final phase C4 with mass of 46.98 g (Figure 3).

### 3.1. Separation Yield

To know the efficiency of our process, we have made a calculation of yield's various crystallization stages according to the following relation:

(1) $$\begin{array}{c} Y=\left(\frac{\left(\text{mass ester}*\text{purity}\right)}{\left(\text{initial mass}*\text{initial purity}\right)}\right)*100. \end{array}$$

**Figure 4:**
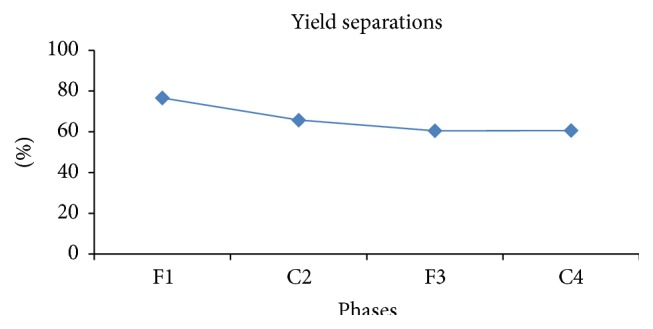
Yield of four crystallizations.

As a result we have found 46.98 g esters with a purity of about 95.49%. We can say that the yield of our method is very satisfying since we could reach a value of 60.61%, and we also see after the calculation of yield of other separations the results are very important because we have had results that exceed 60% (Figure 4).

### 3.2. Saponification Yield

From 150 mL (138 g) of oil we could produce approximately 141.67 g soap whose characteristics are presented in Table 6.
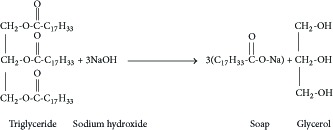
(2)

 We accept as data
 
*M* (oil) = 884 g/mol and *M* (soap) = 304 g/mol
 and *n* (oil) = *n* (soap)/3
 
*m* (oil)/*M* (oil) = *m* (soap)/3 *∗ M* (soap)
 with *m* (oil) = 138 g and *m* (soap) experimental = 141.67 g
 
*m* (soap) theoretical = 3 *∗* 304 *∗* 138/884 = 142.37 g
  (3) $$\begin{array}{c} Y=\left(\frac{m\left(soap\right)exp}{m\left(soap\right)th}\right)*100\\ Y=\left(\frac{141.67}{142.37}\right)*100=99.50\%. \end{array}$$

The yield of saponification was determined to know the efficiency of our process, seeking to improve it more by the optimization of operating conditions; 0.5% yield losses can have various causes: parasitic reactions, losses at the various stages of the synthesis (filtration, drying, and crystallization).

The saponification reaction allowed having a soap light beige color with a clean look smooth to the touch with abundant and consistent foam.

A comparison of the characteristics of soap obtained with those relating to soap made from olive pomace [63] showed that the values are quite comparable.

Glycerol derived from the saponification reaction and the transesterification is a viscous liquid, which is transparent, with a sweet taste whose characteristics are shown in Table 7.

Glycerin is soluble in water and in alcohols, very stable under normal conditions of use, and nontoxic and has no negative impact on the environment.

By comparing our glycerol and that produced by the method described in [63], we observe that both have the same characteristics, even if they do not belong to the same origin.

From 138 g of olive oil we could produce 9.14 g of glycerol by saponification, and from 184 g we were able to get 11.31 g by transesterification.

**Figure 5:**
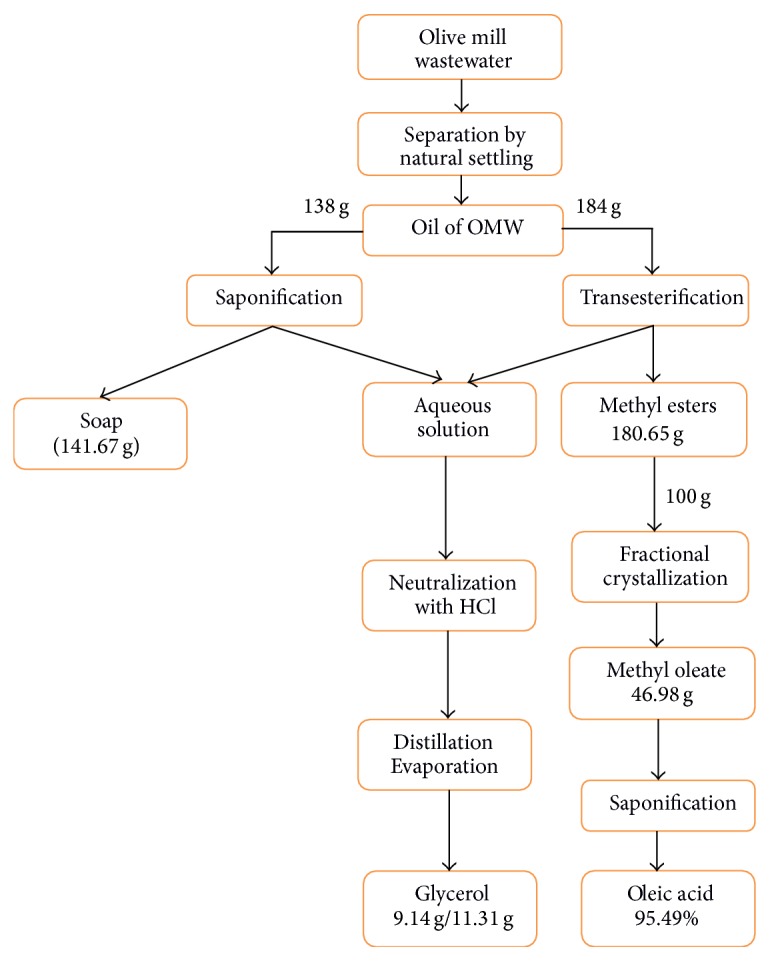
General diagram of the process.

According to the results found in our process, an overall recovery diagram can be proposed (Figure 5).

## 4. Conclusions

In this work, it was possible to recover valuable products from a food waste that causes detrimental effects on nature.

In the first part, we have separated our oil from the olive mill wastewater collected, just by natural decantation. And we could extract oleic fatty acid as a product with a very important commercial value, by fractional crystallization in laboratory scale that we could separate it with a high purity of 95.49%.

The second part of this work was intended to saponify this poor nutritional quality oil to produce good quality soap and for the preparation of glycerin used in several areas.

In this work, we performed a quick and easy process for extracting a very expensive fatty acid market from olive oil wastewater with an easy method Instead of its preparation from olive oil.

## Figures and Tables

**Table 1:** The main physicochemical characteristics of Moroccan olive mill wastewater.

Parameters	Values
pH	4.7
Acidity (%)	1.3
FM (%)	1
SM (g/L)	0.5
EC (mS/cm)	18.7
COD (g O_2_/L)	84.1
BOD_5_ (g O_2_/L)	30
PP (g/L)	0.2
TNK (g N/L)	0.1
Chlorides (g/L)	5.1

FM: fat matter, SM: suspended matter, EC: electrical conductivity, COD: chemical oxygen demand, BOD_5_: biochemical oxygen demand, PP: polyphenols, and TNK: total nitrogen Kjeldahl.

**Table 2:** Source and mass fraction of material.

Material	Source	Purity % mass
Sodium hydroxide	VWR International	98%
Sodium chloride	VWR International	99.5%
Potassium hydroxide	VWR International	85%
Hydrochloric acid	VWR International	37%
Ethanol	VWR International	95%
Methanol	VWR International	99.9%
Hexane	VWR International	95%
Acetone	VWR International	99%
Iodine monochloride	VWR International	98%
Sodium thiosulfate	VWR International	99.50%
Acetic acid	VWR International	99.90%
Potassium iodide	VWR International	99%
Ethyl oxide	VWR International	≥99.5%
Chloroform	VWR International	≥99%
Starch, soluble	VWR International	99.00%
Phenolphthalein	VWR International	99.00%
Urea	VWR International	≥99%
Anhydrous sodium sulfate	VWR International	99.00%

**Table 3:** Characteristics of recovered oil from OMW.

Parameters	Values	Norm Codex	Methods
Acidity (%)	3.397	0.3–1	ISO 660 (determination of acid value and acidity)
Iodine index (g/100 g)	82.17	75–94	ISO 3961 (determination of iodine value)
Peroxide index (meq O_2_/kg)	11.26	≤20–≤15	ISO 3960 (determination of peroxide value)
Saponification index (mg KOH/g)	189	184–196	ISO 3657 (determination of saponification value)
Refractive index (*n*_20_^*d*^)	1.4678	1.4677–1.4705	ISO 6320 (determination of refractive index)

**Table 4:** Evolution of compounds during separation (% mass fraction).

Fatty acids	Structure	Composition	F1	C1	F2	C2	F3	C3	F4	C4
Palmitic acid	C16:0	9.3429	2.1019	38.8984	0.2920	4.2546	1.7856	12.9456	1.0024	2.5164
Palmitoleic acid	C16:1	0.6096	1.6539	0.0357	2.8123	2.2454	2.5475	4.5789	6.6221	1.9015
Stearic acid	C18:0	2.9059	0.0476	13.9547	0.2225	0.1614	—	—	—	—
Oleic acid	C18:1	74.018	82.3594	35.9476	43.881	88.1354	90.1357	80.2536	27.1524	95.495
Linoleic acid	C18:2	10.9884	12.2856	4.8458	46.5568	3.6213	5.4456	2.2219	64.6214	1.5654
Linolenic acid	C18:3	2.1352	1.5516	6.3178	6.2354	1.5819	0.0856	—	0.6017	0.4231

**Table 5:** Characteristics of separated oleic acid.

Parameters	Literature [54]	Values
Density	—	0.898
Boiling point (°C)	—	360
Melting point (°C)	14< <15	13.7

**Table 6:** Characteristics of the resulting soap.

Parameters	Values	Literature [63]	Methods
pH	8.7		
Fatty acid	65.30%	64.60%	ISO 685 (determination of total alkali content and total fatty matter content)
Moisture	5.60%	5.00%	ISO 672 (determination of moisture and volatile matter content)
Combined alkali	8.85%	8.85%	ISO 456 (determination of free caustic alkali)
Chloride	3.70%	3.90%	ISO 457 (determination of chloride content—Titrimetric method)
Free alkali	0.10%	0%	ISO 684 (determination of total free alkali)
Glycerol	0.70%	0.70%	ISO 1066 (determination of glycerol content—Titrimetric method)

**Table 7:** Characteristics of the resulting glycerol.

Parameters	Literature [63]	Values
Density	1.26	1.263
Boiling point (°C)	290	290
Melting point (°C)	17-18	17.5
